# Learn to Steer through Deep Reinforcement Learning

**DOI:** 10.3390/s18113650

**Published:** 2018-10-27

**Authors:** Keyu Wu, Mahdi Abolfazli Esfahani, Shenghai Yuan, Han Wang

**Affiliations:** School of Electrical and Electronic Engineering, Nanyang Technological University, 50 Nanyang Ave, Singapore 639798, Singapore; wukeyu@ntu.edu.sg (K.W.); MAHDI001@e.ntu.edu.sg (M.A.E.); SYUAN003@e.ntu.edu.sg (S.Y.)

**Keywords:** deep reinforcement learning, autonomous steering, depth image

## Abstract

It is crucial for robots to autonomously steer in complex environments safely without colliding with any obstacles. Compared to conventional methods, deep reinforcement learning-based methods are able to learn from past experiences automatically and enhance the generalization capability to cope with unseen circumstances. Therefore, we propose an end-to-end deep reinforcement learning algorithm in this paper to improve the performance of autonomous steering in complex environments. By embedding a branching noisy dueling architecture, the proposed model is capable of deriving steering commands directly from raw depth images with high efficiency. Specifically, our learning-based approach extracts the feature representation from depth inputs through convolutional neural networks and maps it to both linear and angular velocity commands simultaneously through different streams of the network. Moreover, the training framework is also meticulously designed to improve the learning efficiency and effectiveness. It is worth noting that the developed system is readily transferable from virtual training scenarios to real-world deployment without any fine-tuning by utilizing depth images. The proposed method is evaluated and compared with a series of baseline methods in various virtual environments. Experimental results demonstrate the superiority of the proposed model in terms of average reward, learning efficiency, success rate as well as computational time. Moreover, a variety of real-world experiments are also conducted which reveal the high adaptability of our model to both static and dynamic obstacle-cluttered environments.

## 1. Introduction

Autonomous robots have been built for a wide range of applications, such as surveillance, exploration, data collection, inspection, rescue and service, etc. [1]. In most of the missions, it is essential for autonomous robots to detect and avoid various obstacles while maneuver in unknown cluttered environments safely. Numerous methods have been proposed and adapted successfully to different robots [2,3,4,5]. However, conventional methods may impose intensive computational demand [6,7] and are often built upon a set of assumptions that are likely not to be satisfied in practice [8,9]. Moreover, conventional algorithms typically include a number of parameters need to be tuned manually [10] rather than being capable to learn from past experiences automatically [11]. Therefore, it is difficult for these approaches to generalize well to unseen scenarios.

Without any labeled dataset, deep reinforcement learning (DRL) has achieved remarkable success in solving challenging problems in many domains, such as video games [12], continuous control [13], and robotics [14,15]. Recently, the preliminary study of DRL algorithms in the context of autonomous steering has demonstrated that effective control policies can be directly mapped from raw sensor inputs [16,17,18,19]. The network proposed in Ref. [20] output control commands based on ten-dimensional laser range inputs and was trained using an asynchronous DRL algorithm. Similarly, the models introduced in Refs. [21,22] also derived the steering commands from laser range sensors, with the former utilizing the intrinsic curiosity to augment the reward signals while the latter assisting the DRL algorithm using an external controller. Since it is impractical to train DRL algorithms in real-world environments considering the excessive time consumption and heavy workload required for interaction, laser-based methods are competitive in terms of the transferability to real world because of the smaller discrepancies between their simulated and real domains.

Nevertheless, a limitation of these 2D laser-based DRL architectures is the lack of 3D information while 2D sensing data is not informative enough to describe complex 3D scenarios. On the contrary, vision sensors are capable of providing 3D observations to achieve higher reliability and, in the meantime, are generally more affordable as well. Instead of using laser range finders, Zhu et al. proposed a DRL framework to derive control policies for mobile robots directly from RGB images [23] and succeeded in adapting their model to a real robot scenario after fine-tuning. In Ref. [24], the segmented rather than raw RGB images were used while training a DRL model to derive motion commands for humanoid robots. However, it is as expected that RGB inputs suffer from the significant deviation between real-world situations and the synthetic environments during training, which leads to quite limited generalization across situations. Compared to RGB images, the depth inputs in simulations exhibit much better visual fidelity due to the textureless nature and, as a result, greatly alleviate the burden of transferring the trained model to real deployments [25].

Based on raw depth images, Tai et al. trained their network through the Deep Q-Network (DQN) for a mobile robot to avoid obstacles in unknown environments [26], and in Ref. [27], Zhang et al. proposed to use successor features to achieve efficient knowledge transfer across tasks in depth-based navigation. Rather than adopting depth inputs directly, the network presented in Ref. [10] comprised two parts. Firstly, a fully convolutional residual network [28] was implemented to predict depth information from the input monocular RGB images. The estimated depth images were subsequently fed into a dueling architecture based double DQN for action selection. Due to the extraordinary performance of Convolutional Neural Networks (CNNs) in feature extraction [29,30], all the aforementioned depth-based approaches employed CNNs to generate feature representations so that the networks were end-to-end trainable without any explicit extraction of hand-crafted features. Meanwhile, based on CNN architectures, most of these models were trained using the value-based DRL algorithms, including DQN and its variants.

Introduced by Mnih et al. [12,31], DQN has achieved breakthroughs in tackling a variety of challenges, including autonomous steering tasks [32]. Generally, DQN solves problems with high-dimensional observation spaces by leveraging deep neural networks to estimate the Q-values for corresponding actions and taking advantage of two significant innovations. Firstly, DQN adopts the experience replay technique to improve data efficiency. At each step, it updates the deep neural network through randomly sampling a mini-batch of stored experiences from the replay buffer. Besides, it also separates the target network from the behavior network to achieve more stable trainings. As an important milestone, many extensions of DQN have been proposed so far to further improve its performance. The double DQN (DDQN) was proposed to address the overestimation issue of Q-learning by decoupling action selection and evaluation during performing the maximization operation for the bootstrap target [33]. The dueling network architecture introduced in Ref. [34] generalized the learning across actions by explicitly separating the representation of state values and action advantages. The Noisy DQN proposed by Fortunato et al. contributed to enhance the exploration by combining both deterministic and noisy streams in a linear layer [35].

Despite the extraordinary success of DQN in processing high-dimensional state spaces, it is incompetent to cope with continuous action spaces directly and generally requires sampling actions from discrete spaces to simplify the problems. Indeed, although DQN theoretically leads to better sample efficiency compared to policy gradient approaches, its performance is restricted when dealing with high-dimensional discrete action spaces [36]. Therefore, the aforementioned DQN-based methods are quite limited in their allowed ranges of control outputs.

In this paper, we propose a new variant of DQN which can derive control commands effectively from depth images and learn to steer automatically with high efficiency. By embedding a branching noisy dueling architecture in a DDQN, our network, which is named BND-DDQN, combines the merits of multiple variants of DQN and addresses the limitation of DQN in handling actions in continuous domains. Specifically, the feature representation is firstly extracted from raw depth inputs using CNNs. Subsequently, we map it to two Q-value vectors separately via different branches of the network. Based on the estimated Q-values, the optimal linear and angular velocity commands can then be determined and executed simultaneously to improve the response capability of the system. Moreover, a tailored training framework is also proposed to enable the system to learn desired behaviors more efficiently. Compared to previous value-based DRL algorithms, the performance of the proposed model is dramatically improved and, in the meanwhile, the number of selectable control outputs is also increased. It is worth mentioning that although our model is trained in virtual environments, it can adapt to real-world situations readily without any fine-tuning due to the usage of depth information. Experiments have been conducted in both virtual and real-world environments to evaluate the proposed BND-DDQN model. The experimental results demonstrate the superiority of the proposed algorithm and reveal its outstanding transferability to both static and dynamic unknown environments in practice.

The rest of the paper is organized as follows. Section 2 provides a brief introduction of the background and preliminaries. In Section 3, the proposed depth-based DRL algorithm is described in detail. The experimental results are presented and discussed in Section 4. Finally, conclusions are given in Section 5.

## 2. Background and Preliminaries

### 2.1. Reinforcement Learning

Markov Decision Processes (MDPs) provide a mathematical framework to model stochastic planning and decision-making problems under uncertainty [37]. An MDP is a tuple M=(S,A,R,P,γ), where *S* indicates the state space, *A* is the action space, *R* represents the reward function which illustrates the immediate state-action reward signal, *P* indicates the transition function which describes the probability distribution over states if an action *a* is taken in the current state *s*, and γ∈[0,1] is discount factor. In an MDP, a policy π(a|s) specifies the probability of mapping state *s* to action *a*. The superiority of a policy π can be assessed by the action-value function (or Q-value) defined as:

(1) $$Q^{\pi }\left(s,a\right)=E^{\pi }\left[\sum_{t=0}^{\infty }\gamma^{t}R\left(s_{t},a_{t}\right)|s_{0}=s,a_{0}=a\right].$$

Therefore, the action-value function is the expectation of discounted sums of rewards, given that action *a* is taken in state *s* and policy π is executed afterwards. The objective of the agent is to maximize the expected cumulative future reward, and this can be achieved by adopting the Q-learning algorithm which approximates the optimal action-value function iteratively using the Bellman equation described in the following:

(2) $$Q^{*}\left(s_{t},a_{t}\right)=R\left(s_{t},a_{t}\right)+\gamma \max_{a_{t+1}}Q\left(s_{t+1},a_{t+1}\right).$$

### 2.2. Deep Q-Learning

Combined with deep neural networks, DQN enables reinforcement learning to cope with complex high-dimensional environments in which there are a large number of combinations of states and actions [12]. Generally, DQN maintains two deep neural networks, including an online network with parameters θ and a separate target network with parameters $\theta^{-}$. During each iteration, an action is determined using the ϵ-greedy policy. That is, actions are greedily selected most of the time according to the action-value function resulted from the online network while are randomly generated with a probability of ϵ. Subsequently, the new transition after executing the selected action is stored in the experience replay buffer. In the meantime, a random mini-batch of stored transitions is sampled from the replay memory and the estimated optimal action-value function is then updated by performing gradient descent on ${\left(y_{t}-Q\left(s_{t},a_{t};\theta \right)\right)}^{2}$ with respect to parameters θ, where $y_{t}$ can be calculated as follows:

(3) $$y_{t}=\left\{\begin{array}{cc} r_{t} & \mathrm{if}\mathrm{episode}\mathrm{terminates}\mathrm{at}\mathrm{step}t+1\\ r_{t}+\gamma \max_{a_{t+1}}Q\left(s_{t+1},a_{t+1};\theta^{-}\right) & \mathrm{otherwise} \end{array}\right..$$

Thus, the parameters of the online network are updated constantly by minimizing the loss function while the parameters of the target network are fixed for generating Temporal-Difference (TD) targets and synchronized regularly with those of the online network.

The maximum operation, however, introduces a positive bias towards the estimation of Q-values. Therefore, DDQN is proposed to alleviate this overestimation by decoupling the action selection from the generation of action-value [33]. Instead of regarding the maximum Q-value of the target network as the target action-value, DDQN selects the best action for the next state based on the online network while estimates the target action-value through calculating the Q-value of the selected action according to the target network. Therefore, $y_{t}$ is rewritten as Equation (4) in DDQN.

(4) $$y_{t}=\left\{\begin{array}{cc} r_{t} & \mathrm{if}\mathrm{episode}\mathrm{terminates}\mathrm{at}\mathrm{step}t+1\\ r_{t}+\gamma Q\left(s_{t+1},\underset{a_{t+1}}{\mathrm{argmax}}Q\left(s_{t+1},a_{t+1};\theta \right);\theta^{-}\right) & \mathrm{otherwise} \end{array}\right.$$

Moreover, a dueling architecture based DDQN has been introduced as a further extension of DQN [34]. In Dueling DDQN, the network is split into two streams with one providing an estimate of the state value function V(s) and the other calculating the state-dependent action advantages A(s,a). The action-state function is then estimated by combining the two streams through an aggregation layer and can be expressed as:

(5) $$Q\left(s_{t},a_{t};\theta ,\theta_{V},\theta_{A}\right)=V\left(s_{t};\theta ,\theta_{V}\right)+A\left(s_{t},a_{t};\theta ,\theta_{A}\right)-\frac{1}{N}\sum_{a}A\left(s_{t},a;\theta ,\theta_{A}\right),$$

where θ, $\theta_{V}$, $\theta_{A}$ denote the common network parameters, the value stream parameters and the advantage stream parameters, respectively, and *N* represents the total number of actions. In this way, the state values can be obtained without calculating the action-values for all actions, which contributes to a more reliable estimation of Q-values.

## 3. Architecture and Implementation of BND-DDQN

### 3.1. Problem Definition

The objective of our work is to learn the autonomous steering policies effectively and efficiently through training the proposed network architecture using a reinforcement learning algorithm. The designed model is aimed to derive both linear and angular velocity commands directly from the raw depth images in real time. By choosing depth images as the inputs of the neural network, the transferability of our model to real deployments is significantly increased while the full 3D sensing information is also well preserved. Before the introduction of our model, a description of the input state, output action and the designed reward function is presented in the following.

A state of the agent is consisted of a stack of depth images from four consecutive steps to maintain the temporal information. The images are all captured from the first-person view and resized to 80×100. With a series of four depth images as input, our neural network outputs the steering commands by selecting actions from the designed action space. Instead of considering merely a few simple commands such as ‘moving forward’, ‘turning left’, and ‘turning right’, our action space of the agent contains a set of control commands so that both a linear velocity and an angular velocity can be selected separately from the action pool corresponding to the input state. The action space of our agent is meticulously defined so that both linear and angular velocities are executed simultaneously to acquire more stable policies through addressing the problems associated with coarse action discretization.

Although sparse reward functions are much easier to design, they typically slow down the learning since more experiences are required for the sparse signal to propagate throughout the representation of the state space. A dense reward, on the contrary, provides more information for each move to simplify the learning process. Therefore, an informative instantaneous reward function is designed as illustrated in Equation (6), which allows the model to learn safe, fast and smooth steering with high efficiency.

(6) $$r\left(s_{t},a_{t}\right)=\left\{\begin{array}{cc} -10 & \mathrm{if}\mathrm{in}\mathrm{collision}\\ c_{1}v^{2}cos\left(c_{2}v\omega \right)-c_{3} & \mathrm{otherwise} \end{array}\right.$$

In Equation (6), *v* and ω represent the linear and angular velocity, respectively while $c_{1}$, $c_{2}$ and $c_{3}$ are three constant factors that can be tuned according to the specifications of the robotic system. In specific, the first constant $c_{1}$ is a scaling factor and the third constant $c_{3}$ is a bias. These two coefficients are to determine the range of the reward function so that its largest value is approximately equal to one while its distribution is appropriately defined. Besides, the second factor $c_{2}$ is to regulate the effect of angular velocity as the linear velocity changes. According to the definition, the agent is desired to move straight forward as fast as possible and the difference in linear velocity leads to significant difference in reward when the angular velocity is small. By tuning $c_{1}$ and $c_{3}$, the reward is much smaller than −10 when no collision is detected so that the largest penalty is resulted from bumping into an obstacle. As a result, a larger angular velocity will be selected if it is necessary for the agent to change its heading orientation for obstacle avoidance. In these situations, the linear velocity plays a less important role in determining the reward as the angular velocity increases and the attenuation of the effect of linear velocity can be customized by tuning constant $c_{2}$. From a security standpoint, for instance, the reward can even be designed to drop when a large linear velocity is picked together with a large angular velocity. With the defined reward signal, our goal is to maximize the cumulative reward received by the agent in the long run.

### 3.2. Network Architecture

**Figure 1:**
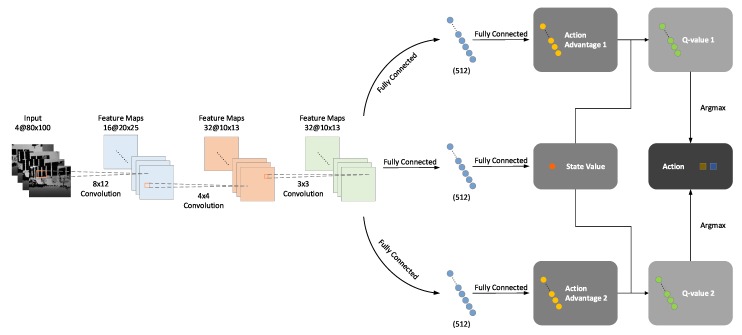
Network architecture of the BND-DDQN model. Input of the network is the state of the agent, which consists of a sequence of depth images from four consecutive steps. The feature representation is generated from these raw depth inputs via three convolutional layers. Subsequently, the network branches into three streams to map the feature representation to one common state value and two action advantages corresponding to linear and angular velocity commands, respectively. The action-value functions are then estimated through aggregation layers and the optimal actions are greedily selected.

In order to achieve the aforementioned objectives, we propose a new deep neural network named BND-DDQN to realize the non-linear approximation of the control policy. Our BND-DDQN model adopts a branching noisy dueling architecture built upon the DDQN framework, and its network structure is illustrated in Figure 1.

As introduced in Section 3.1, a series of four 80×100 depth images obtained from four successive steps are concatenated together as the input of our BND-DDQN model, and three convolutional layers activated by the ReLU function are then used to generate the 4160-D feature representation from the raw depth inputs. Specifically, the first convolutional layer filters the input images with 16 kernels of size 8×12 and a stride of 4 pixels. Next, the second convolutional layer produces 32 feature maps with 4×4 kernels and a stride of 2 pixels. The output is subsequently filtered by the last convolutional layer using 32 kernels of size 3×3 and a stride of 1 to yield 32 feature maps of size 10×13, which are then flattened into a vector and fed to the fully connected layers.

On the whole, the network splits into three streams after the feature representation is obtained. One of the branches is to estimate the state-value function while the other two aim to calculate the action advantages in different dimensions which correspond to linear and angular velocities, separately. More specifically, the 4160-D feature vector is passed into two fully connected layers with 512 neurons and 1 neuron, respectively, to estimate the state value. In the meantime, the advantages of actions associated to linear velocity commands are acquired by feeding the same feature vector through two fully connected layers with 512 and *N* units, respectively, where *N* refers to the number of discretized actions. Similarly, the feature representation is mapped to action advantages corresponding to angular velocities via another two fully connected layers with the same configuration. In each branch, the first fully connected layer is followed by a ReLU activation function while no activation is applied for the second fully connected layer.

It is worth noting that instead of utilizing conventional fully connected layers, our model implements the concept of NoisyNets [35] to achieve more efficient exploration in a more consistent way. This is achieved through perturbing the weights and biases by a parametric function of noise. To begin with, it is known that a linear layer can be expressed as y=ωx+b, where *x* is the input, *y* is the output, ω represents the weight matrix, and *b* denotes the bias. In order to achieve the exploration, the uncertainty can be added by introducing Gaussian noises as shown in Equation (7), where $\mu_{\omega }$, $\sigma_{\omega }$, $\mu_{b}$, and $\sigma_{b}$ are the parameters of the network whereas $\epsilon_{\omega }$ and $\epsilon_{b}$ are random noises, and ⊙ denotes element-wise multiplication.

(7) $$y=\left(\mu_{\omega }+\sigma_{\omega }⊙\epsilon_{\omega }\right)x+\mu_{b}+\sigma_{b}⊙\epsilon_{b}$$

However, the introduction of noise can lead to a large number of noise variables. Therefore, rather than independent noises, factorized Gaussian noises can be used to alleviate the computational burden. The weight matrix can then be re-formatted as:

(8) $$\omega_{i,j}=\mu_{i,j}^{\omega }+\sigma_{i,j}^{\omega }f\left(\epsilon_{i}\right)f\left(\epsilon_{j}\right),$$

and the corresponding bias can be re-written as:

(9) $$b_{j}=\mu_{j}^{b}+\sigma_{j}^{b}f\left(\epsilon_{j}\right),$$

where function *f* is defined as $f\left(\epsilon \right)=\mathrm{sgn}\left(\epsilon \right)\sqrt{\left|\epsilon \right|}$. Besides, the initial values of $\mu^{\omega }$ and $\mu^{b}$ are randomly sampled from the uniform distribution on the interval $\left(-\frac{1}{\sqrt{N}},\frac{1}{\sqrt{N}}\right)$, where *N* is the size of the input layer. Meanwhile, the values of $\sigma^{\omega }$ and $\sigma^{b}$ are initialized to $\frac{0.4}{\sqrt{N}}$.

In this way, the state value V(s) and the two advantages $A_{1}$ and $A_{2}$ are estimated via the noisy fully connected layers and combined thereafter through a special aggregation layer to produce the estimates of the action-value functions $Q_{1}$ and $Q_{2}$ as illustrated in Equation (10), where $N_{i}$ is the size of action space for the *i*-th action dimension.

(10) $$\begin{array}{cc} Q_{i}\left(s,a_{i}\right)=\mathrm{ReLU}\left(V\left(s\right)+A_{i}\left(s,a_{i}\right)-\frac{1}{N_{i}}\sum_{a_{i}^{\prime }}A_{i}\left(s,a_{i}^{\prime }\right)\right), & i=1,2 \end{array}$$

Since the exploration is achieved through the addition of Gaussian noises, the ϵ-greedy strategy is no longer used while interacting with the environments. Instead, the optimal action is greedily chosen according to the learned policy.

### 3.3. Training Framework

**Figure 2:**
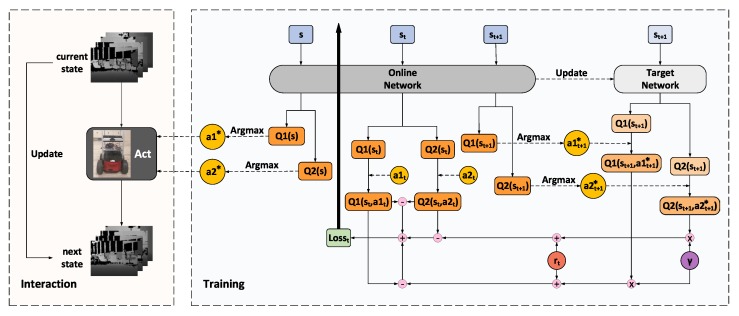
Training framework of the BND-DDQN model. The online network is used to output control commands and evaluate the policies while the target network is employed to estimate the target values for evaluation. At each iteration, the current state is fed through the online network to compute the Q-values of the executed actions whereas the next state is passed into both networks to determine the corresponding target Q-values together with the discounted factor and the immediate reward. The loss function is calculated as a combination of three terms, including the difference between the first Q-value and its target value, the difference between the second Q-value and its target value, and the difference between the two Q-values. The loss is back-propagated through the online network to update its parameters while the target network is a periodic duplicate of the online network.

Built upon the proposed branching noisy dueling architecture, our BND-DDQN model is trained following the protocol depicted in Figure 2. At the beginning, the parameters of the online network θ are initialized randomly while those of the target network $\theta^{-}$ are assigned as a duplication. The online network is subsequently utilized to select optimal actions based on the states of the agent. At each step, the system receives an immediate reward and simultaneously transits to a new state after executing the determined control commands. The new state is then updated to be the input state for determination of the optimal actions in the next step. In the meantime, all these transitions are stored in the experience replay memory which is initialized to a capacity of 1000 before training and updated continuously with a maximum capacity of 30,000.

During training, the online network is used to evaluate the policies while the target network is deployed to estimate the TD target values. A mini-batch of transitions is sampled from the experience replay buffer to update the network at each iteration and these training data are composed of the current state, executed actions, immediate reward, and the next state. Firstly, the current state $s_{t}$ is fed through the online network to calculate the two vectors of action-state values, $Q1(s_{t};\theta )$ and $Q2(s_{t};\theta )$, which correspond to the discretized linear and angular velocity commands, respectively. The Q-values of the executed actions, $a1_{t}$ and $a2_{t}$, are then extracted as $Q1(s_{t},a1_{t};\theta )$ and $Q2(s_{t},a2_{t};\theta )$, accordingly, from the two Q-value vectors. In addition, the next state $s_{t+1}$ is also passed into the online network to acquire the optimal actions which lead to the maximum Q-values at step t+1. Meanwhile, $s_{t+1}$ is fed through the target network as well to compute $Q1(s_{t+1};\theta^{-})$ and $Q2(s_{t+1};\theta^{-})$. Complying with DDQN, the action values of the optimal actions chosen by the online network are determined by the target network. Therefore, with a discount factor of γ and the current reward $r_{t}$, the target values, $y_{1}$ and $y_{2}$, of the action-state value functions at step *t* can be calculated using Equation (11), where i=1,2 denotes the category of the action, ϵ and θ represent the noise variables and parameters of the online network, respectively, while $\epsilon^{-}$ and $\theta^{-}$ indicate those of the target network.

(11) $$y_{i}=\left\{\begin{array}{cc} r_{t} & \mathrm{if}\mathrm{episode}\mathrm{terminates}\mathrm{at}\mathrm{step}t+1\\ r_{t}+\gamma Qi\left(s_{t+1},\underset{ai_{t+1}}{\mathrm{argmax}}Qi\left(s_{t+1},ai_{t+1},\epsilon ;\theta \right),\epsilon^{-};\theta^{-}\right) & \mathrm{otherwise} \end{array}\right.$$

Based on the current Q-values attained by the online network and their corresponding target values computed via Equation (11), we define the loss function as:

(12) $$\begin{array}{cc} L(\theta )=E & [\alpha_{1}{\left(y_{1}-Q1\left(s_{t},a1_{t},\epsilon ;\theta \right)\right)}^{2}\\ & +\alpha_{2}{\left(y_{2}-Q2\left(s_{t},a2_{t},\epsilon ;\theta \right)\right)}^{2}\\ & +\alpha_{3}{\left(Q1\left(s_{t},a1_{t},\epsilon ;\theta \right)-Q2\left(s_{t},a2_{t},\epsilon ;\theta \right)\right)}^{2}], \end{array}$$

where $\alpha_{1}$, $\alpha_{2}$, and $\alpha_{3}$ are three weight coefficients. In Equation (12), the first two terms aim to minimize the differences between the Q-values calculated by the online network and their corresponding target values estimated via the target network. Moreover, since the linear and angular velocity commands are executed simultaneously, these two categories of actions are applied jointly as a whole and, therefore, the difference between the two Q-values extracted from the online network is also minimized.

The loss is back-propagated through the online network to adjust its parameters using the Adam optimizer at each iteration. The pseudo-code of our BND-DDQN algorithm is described in Algorithm 1. It is worth mentioning that in consideration of the branching architecture of our BND-DDQN model, the gradients with respect to the parameters of the convolutional layers are divided by two during the backpropagation. The parameters of the target network, on the contrary, are not trainable. In essence, the target network is a periodic duplicate of the online network so as to avoid rapidly fluctuating estimations of target Q-values. 

**Algorithm 1:** BND-DDQN Algorithm**Input**:batch size $N_{B}$, experience reply buffer *D*, maximum size of replay buffer $N_{R}$, update frequency of target network $N_{T}$, initial parameters of online network θ, initial parameters of target network $\theta^{-}=\theta$

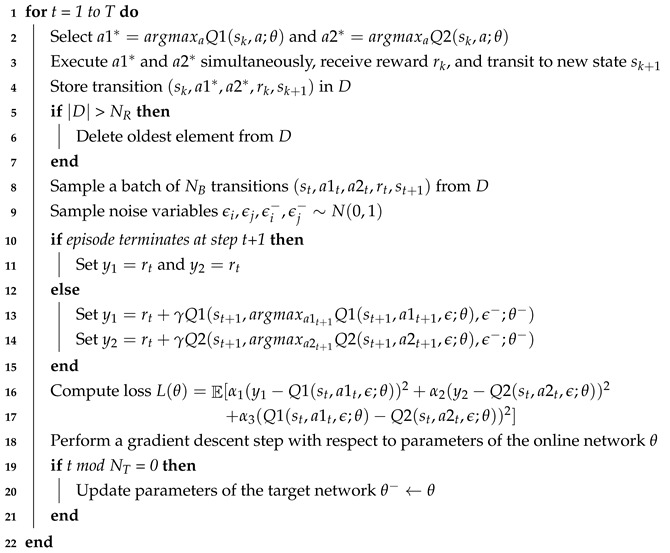



## 4. Experiments and Discussions

### 4.1. Experiments in Virtual Environments

**Figure 3:**
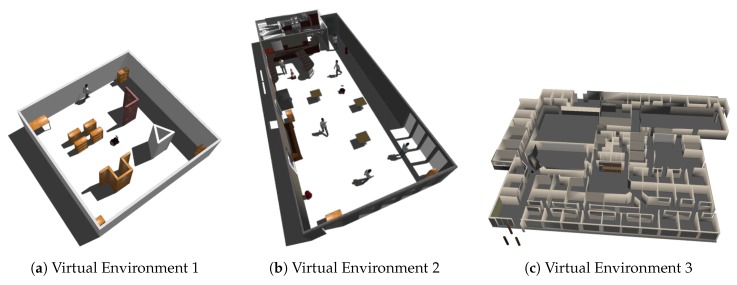
Virtual environments created by Gazebo.

We firstly evaluate our BND-DDQN model and compare its performance with a series of baseline models using a simulated Pioneer 3-AT mobile robot in a variety of virtual environments created by Gazebo [38]. As shown in Figure 3, the first environment is a 10×10 world with relatively larger obstacles whereas the second environment is a more realistic and complicated scenario. Besides, the third environment is the office-like Willow Garage world in gazebo. Our BND-DDQN model is compared with four baseline methods, including DQN, DDQN, Dueling DDQN, and Noisy Dueling DDQN, in all the virtual environments. The input depth images fed into the networks are captured by the depth sensor of a simulated Kinect, and the output control commands are sent to the robot via a ROS message. Besides, the reward function is calculated based on the subscribed odometry message during training.

In the first scenario, all models are trained from scratch with Tensorflow [39] on a single NVIDIA GeForce GTX 1080 Ti with a batch size of 64 and a discount factor γ of 0.99. Rather than decaying the learning rate during training, we adopts a small fixed learning rate of 1e-5. For models achieving exploration through the ϵ-greedy policy, the initial value of ϵ is set to 0.1 and eventually decayed uniformly to 0.0001 after 200,000 iterations. In the second and third virtual environments, the models are initialized via copying weights trained in the first environment. The training is terminated after 5e5 iterations in the first virtual scenario whereas is terminated after 5e4 steps in the other two environments. In all experiments, the constant coefficients of the reward function, $c_{1}$, $c_{2}$ and $c_{3}$, are set to 2, 2, 0.1, respectively. In addition, the maximum number of steps is set to 500, which means an episode is terminated without any punishment after 500 steps. Besides, the weights in the loss function of our model, $\alpha_{1}$, $\alpha_{2}$ and $\alpha_{3}$, are set to 0.4, 0.4, 0.2, correspondingly. The linear velocity is discretized to seven values, including 0.7, 0.6, 0.5, 0.4, 0.3, 0.2, and 0.1 m/s, while the angular velocity is discretized to π/4, π/6, π/12, 0, −π/12, −π/6, −π/4 rad/s. For each baseline model, either a linear or an angular velocity command is picked at each time step, however, both a linear velocity and an angular velocity are selected and executed simultaneously at every step when our BND-DDQN model is employed. Moreover, the starting location in the first environment is the center of the world while in the other two scenarios, the robot starts from a random collision-free location at the beginning of each episode. In all scenarios, the starting orientation of the mobile robot is randomly sampled from the uniform distribution between −π and π rad.

**Figure 4:**
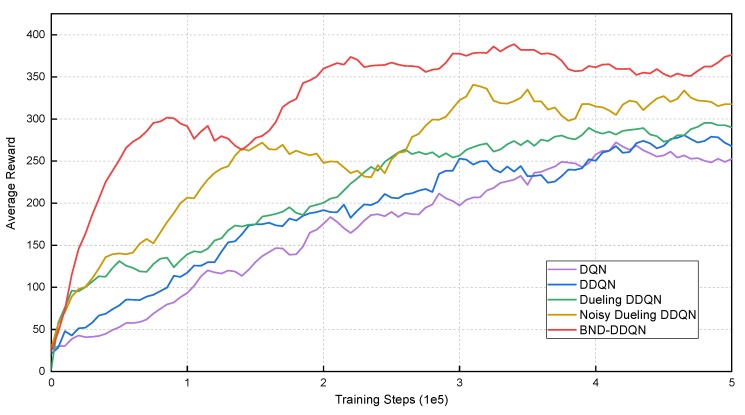
Average total rewards received by various models at different training steps in the first virtual environment. Compared to baseline methods, the BND-DDQN model results in the highest average reward as well as the fastest convergence speed.

During training, the models are evaluated after every 5000 iterations by calculating the average total reward of five episodes. The total reward of an episode is defined as the accumulation of instantaneous rewards received at all steps within that episode, and the average total rewards received by the five models in the first environment are illustrated in Figure 4. It can be observed that the DQN algorithm leads to the lowest average reward at the end of training. The DDQN model performs slightly better than the DQN, nevertheless, results in lower average rewards compared to the Dueling DDQN algorithm. By introducing the noise variables for exploration enhancement, the performance of Noisy Dueling DDQN model is distinctly improved both in terms of average reward and convergence rate. However, the competence of the Noisy Dueling DDQN is inferior compared with the proposed BND-DDQN model. In general, the BND-DDQN algorithm results in the most outstanding performance throughout the evaluation. Through adopting the proposed branching noisy dueling architecture, the BND-DDQN model further speed up the training process and converges in much less number of iterations. In the meantime, our BND-DDQN algorithm also receives significantly increased average rewards.

**Figure 5:**
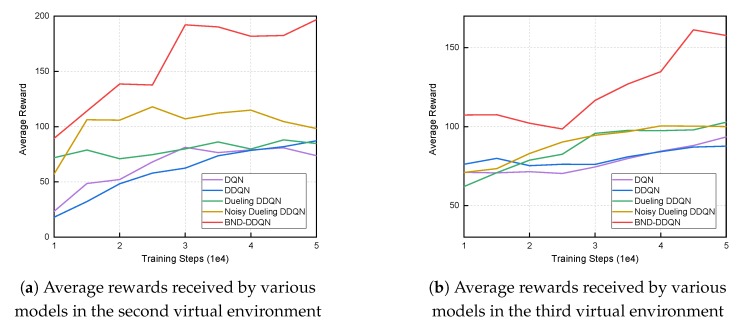
Average total rewards received by various models at different training steps in the second and third virtual environments. The BND-DDQN model results in the highest average rewards compared to baseline methods.

The models are subsequently trained in the second and third environments with parameters initialized via copying the values obtained in the previous stage. Similarly, the evaluation is conducted every 5000 iterations and the average reward received by the five models at different training iterations are demonstrated in Figure 5. Since the situations involved in these two environments are much more challenging compared to those contained in the first scenario, there is a dramatic reduction in acquired average reward. Specifically, in the second environment, the DQN, DDQN and Dueling DDQN algorithms exhibit similar performance and end up with average total rewards of approximately 80. The Noisy Dueling DDQN model reaches an average reward that is around 25 percent higher. It is noteworthy that the proposed BND-DDQN model results in an average total reward of almost 200 at the end of training, which is twice as high as that obtained by the Noisy Dueling DDQN algorithm. Analogously, in the third scenario, our BND-DDQN model is consistently considerably superior compared to the four baseline methods. The baseline algorithms result in similar average rewards with the Dueling DDQN and Noisy Dueling DDQN models performing slightly better than the other two algorithms. Therefore, the proposed BND-DDQN model outperforms the baseline algorithms significantly and its superiority can be more pronounced in challenging scenarios.

In addition to average reward, a comparison is also carried out with respect to success rate. An episode is regarded to be successful if it is terminated with no punishment. During this evaluation, the maximum step of an episode is set to 500 in the first virtual environment whereas is reduced to 200 in the second and third scenarios considering their complexity. In each environment, the success rate of a model is calculated based on the results of 50 episodes and the results are shown in Table 1.

It is noticed that the BND-DDQN model yields the highest success rates in all three cases. In the first environment, all models result in higher success rates due to the simplicity of encountered situations. The lowest success rates are acquired by the DQN and DDQN algorithms while the BND-DDQN model succeeds in all 50 episodes. The superiority of our model become more significant in the second virtual environment. A success rate of 88 percent is obtained by the BND-DDQN model while all the baseline methods result in success rates below 46 percent. Due to the competence of the proposed branching noisy dueling architecture, our BND-DDQN model is capable of taking more effective actions through outputting linear and angular velocity commands simultaneously and, at the same time, can also be trained more efficiently. These features improves the response capability as well as self-learning ability of the system and, hence, plays a vital role in performance improvement, especially in complex changing environments. In the third environment, the success rate of the BND-DDQN model is still much higher than those obtained by baseline algorithms, however, it decreases to 62 percent. The less satisfactory performance of the BND-DDQN model can be caused by several reasons. Firstly, the field of view of the sensor is relatively small and as a result, the depth inputs are not informative enough to derive the desired steering commands in some situations, especially in quite constrained places. More importantly, since merely four images from successive steps are concatenated together as the input of the network at each time step, the sensing information contained in the set of images can be insufficient to provide a reliable description of the surrounding environment, and consequently leads to failures. The problem can be alleviated by modifying the network architecture to take account of more past memories in an efficient way so that more knowledge can be exploited when making decisions. Besides, the instantaneous reward signal as well as the discretized commands can be redesigned to improve the performance of the system.

**Figure 6:**
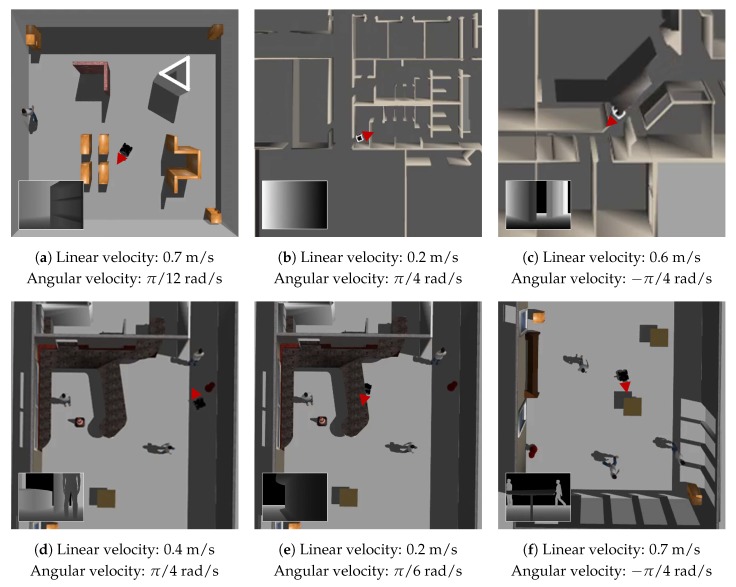
Sample velocity commands produced by the BND-DDQN model based on the estimated Q-values in various scenarios. In each example, the orientation of the mobile robot is highlighted using a red triangle and the raw depth image is shown in the bottom left corner correspondingly.

In order to illustrate the effectiveness of the proposed algorithm in a more intuitive way, six examples of the velocity commands produced by the BND-DDQN model based on the estimated Q-values are demonstrated in Figure 6. In each example, the orientation of the robot is indicated using a red triangle and the corresponding raw depth image acquired from the Kinect is displayed in the bottom left corner. In the first case, a small angular velocity is selected to prevent the mobile robot from colliding with the bookshelf. Since the distances from the robot to the obstacles are still relatively large, the maximum linear velocity is executed. In the much more constrained space shown in the second example, the linear velocity is reduced to only 0.2 m/s and the largest angular velocity is chosen for effective collision avoidance while the robot is passing through a door. A similar scenario is presented in the fifth example. However, since the situation is less imperative, the BND-DDQN model selects a smaller angular velocity in this case. In the third example, the robot is controlled to maintain a moderate linear velocity while execute the maximum angular velocity so as to pass through the corridor. Finally, a positive angular velocity is sent to the robot to avoid a person in the fourth scenario while a negative angular velocity command is selected to prevent the robot from bumping into a table in the last scenario.

Last but not least, it is worth mentioning that the average time required by the BND-DDQN model to derive a pair of control commands from the depth images is merely 0.005 s. Among the baseline algorithms, the DQN and DDQN models, which result in the least computational time, take about 0.002 s. Although the computational cost of our model is higher than the baseline algorithms due to the increase in network complexity for performance improvement, it has already been efficient enough for common mobile robots which typically receive commands at a frequency of tens of Hz for real-time autonomous steering tasks.

### 4.2. Experiments in Real-World Environments

A variety of real-world experiments are conducted as well to verify the proposed BND-DDQN model. In all experiments, we obtain depth images from a Kinect and derive the control commands directly from the depth inputs based on the Q-values estimated by the BND-DDQN model. The steering commands are then sent to a Pioneer 3-AT mobile robot to execute. It is worth noting that the BND-DDQN model trained in the virtual environments is directly transferred to the real-world deployment without any fine-tuning of parameters, and it is proved that the our model is capable of adapting well to real-world unseen scenarios by adopting depth images as the inputs to the network.

**Figure 7:**
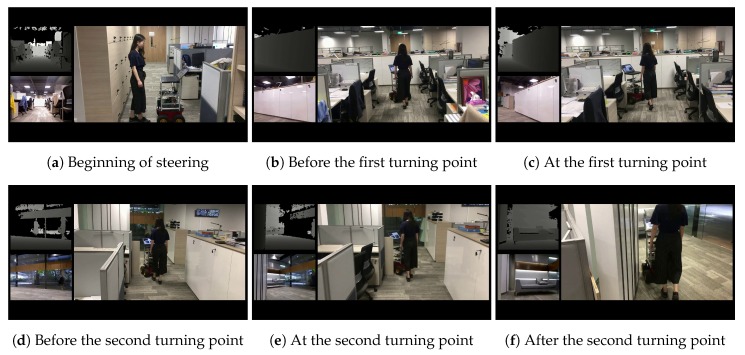
Intermediate steps of the first real-world experiment. In this experiment, the BND-DDQN model is evaluated in a cluttered office environment. For each step, the third-person view is shown on the right and the corresponding depth image and first-person view are displayed in the top and bottom left corners, respectively.

Firstly, our BND-DDQN model is evaluated in a cluttered office environment and six intermediate steps are presented in Figure 7. For each step, the third-person view is provided on the right while the corresponding depth image and first-person view are shown in the top and bottom left corners, respectively. The robot is controlled to travel forward at the beginning to pass through the corridor and larger angular velocities are then selected when the first turning point is detected. Similarly, the robot continues to move forward after the first turning point is passed and starts to receive large angular velocity commands again when the second turning point is identified. By following the control commands provided by our BND-DDQN model, the mobile robot succeeds in achieving safe autonomous steering in the cluttered environment, which demonstrates the effectiveness and high transferability of the proposed algorithm.

**Figure 8:**
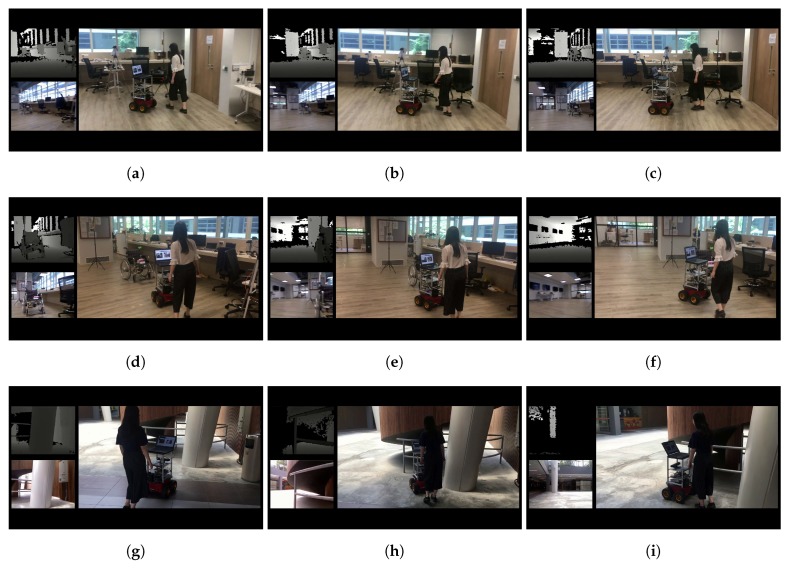
Examples in static environments. In each figure, the third-person view is shown on the right while the corresponding depth image and first-person view are displayed in the top and bottom left corners, respectively. (**a**–**c**) Illustrate the process to avoid colliding with a tripod. (**d**–**f**) Describe the process to avoid bumping into a wheelchair. (**g**–**i**) Demonstrate the process to avoid running into a railing.

In addition to the office environment, the BND-DDQN model is also evaluated in a number of other unseen environments, including both indoor and outdoor scenarios. Three examples in static environments are described in Figure 8. For each example, three steps are demonstrated and, for each step, the third-person view is displayed on the right while the corresponding depth image and first-person view are provided in the top and bottom left corners, respectively. As illustrated in Figure 8a–c, the robot travels towards a tripod at the beginning of the first example. However, after the tripod is perceived, the maneuver direction of the robot is changed by following the velocity commands provided by the BND-DDQN model so that the robot is prevented from bumping into the tripod. Similarly, the control commands derived from the learned policy enables the robot to change its steering direction to avoid a wheelchair in the second example shown in Figure 8d–f, as well as a railing in the third example illustrated in Figure 8g–i.

**Figure 9:**
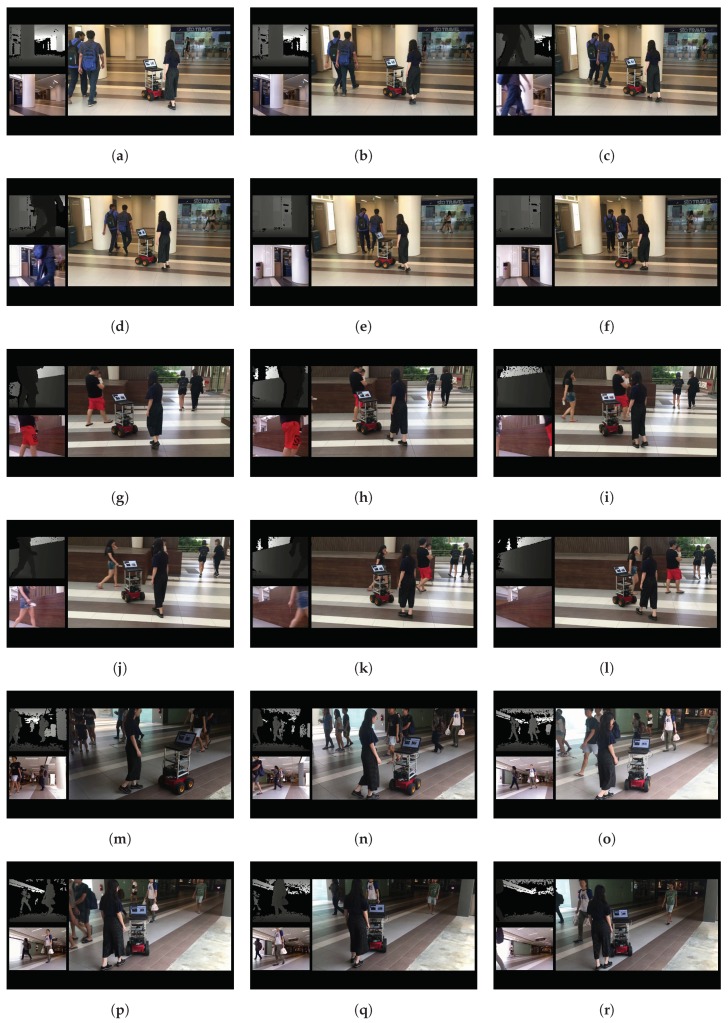
Examples in dynamic environments. For each example, six intermediate steps are demonstrated in sequence. For each step, the third-person view is shown on the right and the corresponding depth image and first-person view are displayed in the top and bottom left corners, respectively. (**a**–**f**) Show the process to avoid collision with two walking persons. (**g**–**l**) Describe the process to avoid a group of people separately. (**m**–**r**) Illustrate an example of traveling through a crowd.

Furthermore, our BND-DDQN model is verified in dynamic environments as well and three examples are demonstrated in Figure 9. For each example, six intermediate steps are presented in sequence, and for each step, the depth image and first-person view are displayed in the top and bottom left corners, respectively, while the corresponding third-person view is presented on the right. In the first example shown in Figure 9a–f, the robot is passing through two pillars at the beginning. However, the learned policy guides the robot to turn left after the two walking persons are detected in the depth images. In this way, the robot is able to continue the steering safely without bumping into the two walking persons. Moreover, in the second example illustrated in Figure 9g–l, the robot is maneuvering towards the stage at first and is subsequently commanded to turn right after a walking person is detected on the left side of the depth image. However, since the detected person is moving from the left side to the right side of the depth image, the robot is controlled to maneuver through the two persons instead of turning to the right. At a later step, the robot is commanded to steer left to avoid colliding with the second pedestrian. In addition, an example of controlling the robot to pass through a crowd based on the learned policy is demonstrated in Figure 9g–l. In this example, the robot is commanded to turn left or right based on the input depth images so as to avoid bumping into people while moving forward.

Besides the aforementioned experiments, more real-world examples can be found in our video at https://youtu.be/yixnmFXIKf4. The experimental results demonstrate that our BND-DDQN model which is trained in the virtual environments can be directly transferred to various real-world unseen scenarios without any fine-tuning.

## 5. Conclusions

It is challenging for conventional methods to generalize well to unseen scenarios because they are often built upon a set of assumptions and typically contain a number of parameters to be tuned manually. Moreover, they are unable to learn the control policies automatically from past experiences. In this paper, we propose an end-to-end deep neural network named BND-DDQN to derive control commands directly from depth images through deep reinforcement learning. The proposed model embeds a branching noisy dueling architecture and is capable of learning autonomous steering with high efficiency. Firstly, we adopt convolutional neural networks to extract features from the depth inputs. The feature representation is then mapped to two Q-value vectors separately through different streams of noisy fully connected layers and aggregation layers. In this way, linear and angular velocity commands can be determined and executed simultaneously to improve the response capability of the system. Moreover, the training framework is also meticulously designed to improve the efficiency and effectiveness of learning. It is worth noting that the proposed model trained in virtual environments can adapt to real-world unseen scenarios readily without any fine-tuning due to the high visual fidelity of simulated depth images.

The BND-DDQN model is evaluated and compared to a series of baseline methods in various virtual environments and the experimental results illustrate the outstanding performance of the proposed algorithm in terms of average reward, learning efficiency, success rate as well as computational time. Furthermore, the proposed model is also evaluated in a variety of real-world environments, including both indoor and outdoor scenarios. The experiments show that our BND-DDQN model is competent to derive effective steering commands in both static and dynamic environments, which demonstrates its high transferability as well as generalization capability. In the future, the architecture of the network will be modified to exploit more knowledge extracted from the past trajectory memories to achieve higher success rate in complex environments.

## Figures and Tables

**Table 1:** Comparison between the proposed BND-DDQN model and four baseline algorithms with respect to success rate in various virtual environments.

Scenario	DQN	DDQN	Dueling DDQN	Noisy Dueling DDQN	BND-DDQN
Virtual Environment 1	84%	84%	88%	92%	**100%**
Virtual Environment 2	26%	30%	30%	46%	**88%**
Virtual Environment 3	34%	36%	42%	40%	**62%**
